# Brillouin Interaction between Two Optical Modes Selectively Excited in Weakly Guiding Multimode Optical Fibers

**DOI:** 10.3390/s23031715

**Published:** 2023-02-03

**Authors:** Andrei Fotiadi, Edik Rafailov, Dmitry Korobko, Patrice Mégret, Alexander Bykov, Igor Meglinski

**Affiliations:** 1 Optoelectronics and Measurement Techniques Unit, University of Oulu, 90570 Oulu, Finland; 2 Electromagnetism and Telecommunication Department, University of Mons, B-7000 Mons, Belgium; 3 Optoelectronics and Biomedical Photonics Group, School of Engineering and Applied Science, Aston University, Birmingham B4 7ET, UK; 4 S.P. Kapitsa Scientific Technological Research Institute, Ulyanovsk State University, 42 Leo Tolstoy Str., 432970 Ulyanovsk, Russia

**Keywords:** multimode optical fiber, stimulated Brillouin scattering, optical fiber amplifiers, mode-division multiplexing, Brillouin imaging, distributed Brillouin sensing

## Abstract

A multimode optical fiber supports excitation and propagation of a pure single optical mode, i.e., the field pattern that satisfies the boundary conditions and does not change along the fiber. When two counterpropagating pure optical modes are excited, they could interact through the stimulated Brillouin scattering (SBS) process. Here, we present a simple theoretical formalism describing SBS interaction between two individual optical modes selectively excited in an acoustically isotropic multimode optical fiber. Employing a weakly guiding step-index fiber approach, we have built an analytical expression for the spatial distribution of the sound field amplitude in the fiber core and explored the features of SBS gain spectra, describing the interaction between modes of different orders. In this way, we give a clear insight into the sound propagation effects accompanying SBS in multimode optical fibers, and demonstrate their specific contributions to the SBS gain spectrum.

## 1. Introduction

Stimulated Brillouin scattering (SBS) is a nonlinear effect that causes light reflection due to interaction with the acoustic phonons. In this process, a narrow-band forward propagating pump wave interferes with a backward Stokes wave (i.e., the wave frequency shifted to the Stokes-side relative to the pump wave), resulting in the moving interference pattern that generates the resonant acoustic waves through the electrostriction. These acoustic waves further cause material density (and refractive index) modulations that, in turn, increase the reflection. In this way, a positive feedback process is created, leading to a drastic increase in the energy conversion from the pump to the Stokes wave. SBS is embedded today in various optical systems, such as advanced low-noise lasers, distributed fiber sensors, microwave signal processors, scientific instrumentation, and optomechanical systems [1].

Some of these applications are tied to particular attributes of SBS in single-mode optical fibers: narrow-band optical amplification, linewidth narrowing, random and narrow-band lasing, controllable light coherency, optical signal processing, etc. The use of multimode fibers as SBS media extends these functionalities, engaging the effects of nonlinear optical mode conversion and optical phase conjugation [2,3] and leading to a new generation of high-performance fiber devices. Brillouin-distributed sensing based on multimode fibers enables the optical Vernier effect [4] that relies on the use of several similar sensors with slightly detuned properties, providing a significant magnification of the sensing capabilities, e.g., by magnifying the measured Brillouin frequency shift compared to a classical BOTDA system [5]. Recently, Brillouin imaging (BI) has become a valuable tool for micromechanical material characterization, thanks to extensive progress in optical fiber instrumentation [6,7]. This powerful technique is contactless and label-free, making it especially suitable for biomedical applications [8]. Randomized light fields [9,10] also open up new forms of optical imaging based on Brillouin scattering. A standard multimode optical fiber provides randomized light propagation, whereas random lasing is available through the Rayleigh–Brillouin cooperative process [11].

There has also been significant progress in modern sensing and imaging techniques that point to miniaturization technologies based on multimode optical fibers. A standard multimode optical fiber can be used as a general-purpose spectrometer after calibrating the wavelength-dependent speckle patterns produced by interference between the guided modes of the fiber [12,13]. Multimode fiber endoscopes with minimal invasiveness are developed for in vivo applications such as 3D imaging, mechanical mapping, ablation of cancerous cells, intraoperative monitoring and optogenetic cell stimulation. They do not require any optical or electro-mechanical elements on the distal fiber end, and can deliver three-dimensional information without pixelation by exploiting wavefront shaping. High-frequency real-time ultrasound imaging [14] can provide exquisite visualizations of tissue to guide minimally invasive procedures. With this device, broad-bandwidth ultrasound generation is achieved through the photoacoustic excitation of a special composite coating on the distal end of the multimode optical fiber by a pulsed laser [15]. Although most commercial sensing systems rely on measurements of the transmitted or reflected fundamental mode of single-mode optical fibers, more recent developments have focused on multimodal architectures that considerably widen the sensing modalities, especially in the chemical and biological fields [16,17,18,19,20]. Mode–division multiplexing is mooted to address the possibility of multiparameter sensing with a single device, the reduction of cross-sensitivities, and the improved accuracy of a single measured parameter by combining the responses of many fiber modes to its evolution. Current progress in mode–division multiplexing relies on the elaboration of new tools for encoding and de-encoding the information stored in the spatial modes of fibers [21,22]. Stimulated Brillouin scattering (SBS) is able to assist all these new paradigms, enabling selective mode amplification, mode conversion and inter-mode signal processing to be implemented immediately inside the multimode optical fibers.

**Figure 1:**
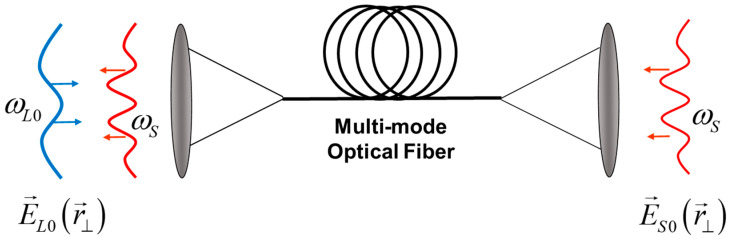
Illustration of Brillouin amplification process in multimode optical fiber. The optical fields with specific profiles ${\vec{E}}_{L0}\left({\vec{r}}_{⊥}\right)$ and ${\vec{E}}_{S0}\left({\vec{r}}_{⊥}\right)$ at frequencies $\omega_{L}$ and $\omega_{S}$ are introduced into the multimode optical fiber, providing selective excitation of pure pump ${\vec{e}}_{L}\left({\vec{r}}_{⊥}\right)\exp\left\{i\left[\omega_{L}t-\beta_{L}z\right]\right\}$ and Stokes ${\vec{e}}_{S}\left({\vec{r}}_{⊥}\right)\exp\left\{i\left[\omega_{S}t+\beta_{S}z\right]\right\}$ optical modes. Their interaction inside the optical fiber with the sound wave leads to amplification of the Stokes mode amplitude.

Stimulated Brillouin scattering in multimode fibers has been previously investigated in a number of works, mainly in the context of the optical phase conjugation effect [2,3,23,24], laser beam combining and cleanup [25]. In particular, the earliest experiments have demonstrated significant difference in the SBS gain factors measured with the optical modes of different orders excited in the same optical fiber sample [26]. The efficiency of the stimulated Brillouin scattering (SBS) process in multimode optical fibers is largely governed by the spatial overlap between the supported optical and acoustic modes leading to a complicated amalgamation of photon–phonon interactions in multimode fibers [27]. Here, we present theoretical formalism to describe SBS dynamics in multimode optical fibers using the weakly guiding step-index optical fiber approach. In contrast to previous studies [28,29,30,31,32,33,34], we consider a simplified situation in which the optical fiber is acoustically uniform and only two counter-propagating pure optical modes are excited and interact inside the fiber, as shown in Figure 1. Interaction between these modes is characterized by an SBS gain spectrum inherent to the interacting mode pair. We have managed to build an analytical expression for the spatial distribution of the sound wave amplitude over the fiber core, and highlight the features of the SBS gain spectrum specifically for interaction between modes of different orders. In this way, we give a clear insight into the sound propagation effects accompanying SBS in multimode optical fibers and demonstrate their specific contributions to the SBS gain spectrum, particularly to the spectrum broadening and splitting in the case of high-order mode interaction. For better understanding of the explored mechanisms, the effects obtained for SBS in optical fiber are compared with similar effects obtained for SBS in a volume medium and planar waveguide.

## 2. Steady-State SBS Model

Let us consider an arbitrary optical fiber (or waveguide) with the input monochromatic pump and Stokes fields shown in Figure 1. We assume that these complex fields are shaped by spatial light modulators and injected into the multimode fiber to excite a pair of pure single eigenmodes. The pump frequency $\omega_{L}$ is fixed, whereas the frequency of the Stokes wave $\omega_{S}$ is tunable. The input pump signal at $\omega_{L}$ excites the eigenmode ${\vec{e}}_{L}\left({\vec{r}}_{⊥}\right)\exp\left\{i\left[\omega_{L}t-\beta_{L}z\right]\right\}$ with propagation constant $\beta_{L}$, and the input Stokes signal at $\omega_{S}$ excites the eigenmode ${\vec{e}}_{S}\left({\vec{r}}_{⊥}\right)\exp\left\{i\left[\omega_{S}t+\beta_{S}z\right]\right\}$ with propagation constant $\beta_{S}$ in a backward direction. It is convenient to characterize the Stokes wave frequency using its dimensionless detuning frequency $\delta =\left(\omega_{S}-\omega_{S0}\right)T_{2}$, where $\omega_{S0}=\omega_{L}-\Omega_{0}$, $\Omega_{0}=2\upsilon n/c$, n is the waveguide core refractive index, c is the velocity of light, υ is the sound wave velocity, and $T_{2}$ is the sound relaxation time [35]. So, the value δ=0 is the resonant SBS frequency shift corresponding to the interaction between two strictly counterpropagating optical plane waves in the volume medium with the same parameters.

To describe steady-state Brillouin amplification of the Stokes mode in the field of the given pump mode, we express the pump, Stokes, and sound wave fields as:

(1) $$\begin{array}{l} {\vec{E}}_{L}\left({\vec{r}}_{⊥},z\right)=A_{L}\cdot {\vec{e}}_{L}\left({\vec{r}}_{⊥}\right)\exp\left\{i\left[\omega_{L}t-\beta_{L}z\right]\right\}\\ {\vec{E}}_{S}\left(\delta ,{\vec{r}}_{⊥},z\right)=A_{S}\left(\delta ,z\right)\cdot {\vec{e}}_{S}\left({\vec{r}}_{⊥}\right)\exp\left\{i\left[\left(\omega_{S0}+\frac{\delta }{T_{2}}\right)t+\beta_{S}z\right]\right\}\\ P\left(\delta ,{\vec{r}}_{⊥},z\right)=A_{L}\cdot A_{S}{}^{*}\left(\delta ,z\right)\cdot p\left(\delta ,{\vec{r}}_{⊥}\right)\exp\left\{i\left[\left(\Omega_{0}-\frac{\delta }{T_{2}}\right)t-\left(\beta_{L}+\beta_{S}\right)z\right]\right\} \end{array}$$

Here, $A_{L}$, $A_{S}\left(\delta ,z\right)$ and $A\cdot B*\left(\delta ,z\right)\cdot p\left(\delta ,{\vec{r}}_{⊥}\right)$ are the complex amplitudes of the interacting fields; $p\left(\delta ,{\vec{r}}_{⊥}\right)$ describes the distribution of the sound wave amplitude in the fiber cross-section, ${\vec{r}}_{⊥}=\left(x,y\right)=\left(r,\varphi \right)$ is the transvers fiber cross-section vector (to be described in Cartesian or cylindrical coordinates), and *z* is the coordinate along the fiber.

Near the resonance, the complex amplitude of the Stokes wave exhibits amplification in backward direction along z:

(2) $$A_{S}(\delta ,z)=A_{S}(\delta ,L)\exp\left\{G\left(\delta \right)\frac{g_{0}}{2}\frac{P_{L}}{S}\left(L-z\right)\right\}$$

where $P_{L}=A_{L}A_{L}{}^{*}$ is the pump power, L is the fiber length, S is the fiber core cross-section area $S=\iint_{core}dS=\iint_{core}rdrd\varphi =\iint_{core}dxdy$, and $g_{0}$ is the SBS power gain factor.

The normalized gain factor $G\left(\delta \right)$ reads as:

(3) $$G\left(\delta \right)=\frac{1}{{\hat{N}}_{L}{\hat{N}}_{S}}\iint_{core}p\left(\delta ,{\vec{r}}_{⊥}\right)\left({\vec{e}}_{L}\left({\vec{r}}_{⊥}\right)\cdot {\vec{e}}_{S}^{*}\left({\vec{r}}_{⊥}\right)\right)dS$$

where ${\hat{N}}_{i}=\iint_{core}\left({\vec{e}}_{i}\left({\vec{r}}_{⊥}\right)\cdot {\vec{e}}_{i}^{*}\left({\vec{r}}_{⊥}\right)\right)dS$ are mode power normalization constants. It is worth noting that ReGδ describes the SBS gain spectrum.

Using Equations (1)–(3), the steady-state SBS problem [36] is reduced to the equation describing the cross-section profile of the acoustic wave amplitude $p\left(\delta ,{\vec{r}}_{⊥}\right)$:

(4) $$\left(1+i\left(\delta -\delta_{LS}\right)\right)p\left(\delta ,{\vec{r}}_{⊥}\right)-i\mu \;\nabla_{⊥}p\left(\delta ,{\vec{r}}_{⊥}\right)=\left({\vec{e}}_{L}^{*}\left({\vec{r}}_{⊥}\right)\cdot {\vec{e}}_{S}\left({\vec{r}}_{⊥}\right)\right)$$

where $\mu =v^{2}T_{2}/2\Omega_{0}$ and $\delta_{LS}=T_{2}\left(\Omega_{0}-v\left(\beta_{L}+\beta_{S}\right)\right)$.

In the next sections, we will solve Equation (4) to compare the SBS interaction between two eigenmodes excited in a volume medium, a planar waveguide and weakly guided step-index fiber. These results expose the sound propagation effects accompanying SBS and highlight their contributions to the SBS gain spectrum and sound wave profile. Without loss of generality, for illustration of the results obtained, we will perform calculations assuming that the optical material is pure silica; the optical waveguide/fiber has a waveguide parameter V~55, the parameter is μ~0.0009, the waveguide/fiber core size/diameter is ~ 50 µm and the laser operation wavelength is ∼ 1064 nm. It is worth noting that the last two parameters are used just to estimate the interaction angles $\alpha_{L}$ and $\alpha_{S}$.

## 3. Comparing SBS Interaction in a Volume Medium and Planar Waveguide

### 3.1. SBS Interaction between Two Plane Waves in a Volume Medium

In a volume medium, the eigen optical modes ${\vec{e}}_{L}\left({\vec{r}}_{⊥}\right)$, ${\vec{e}}_{S}\left({\vec{r}}_{⊥}\right)$ are degenerated to the plane waves expressed as:

(5) $$\begin{array}{l} {\vec{e}}_{L}\left({\vec{r}}_{⊥}\right)=\exp\left[-i\left({\vec{k}}_{L}\cdot {\vec{r}}_{⊥}\right)\right]\\ {\vec{e}}_{S}\left({\vec{r}}_{⊥}\right)=\exp\left[-i\left({\vec{k}}_{S}\cdot {\vec{r}}_{⊥}\right)\right] \end{array}$$

where ${\vec{k}}_{L}$ and ${\vec{k}}_{S}$ are wavevectors of the pump and Stokes plane waves with $\left|{\vec{k}}_{L,S}\right|=\frac{n}{c}\omega_{L,S}$.

**Figure 2:**
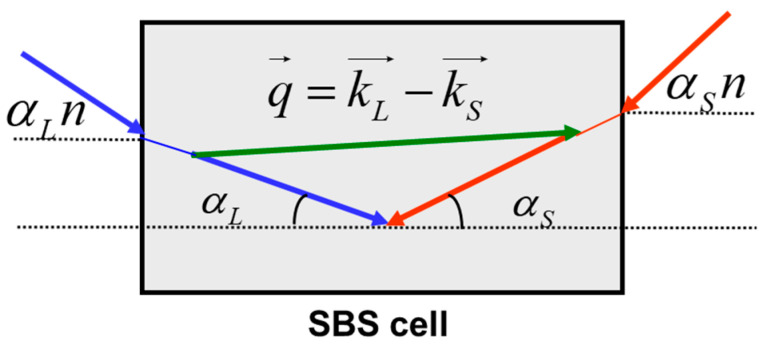
Brillouin interaction between plane waves in a volume medium.

Such plane waves can be defined by setting the angles $\alpha_{L}$ and $\alpha_{S}$ between the corresponding wave vector (${\vec{k}}_{L}$ or ${\vec{k}}_{S}$) and the axis z. Figure 2 illustrates the process of the pump–Stokes wave interaction in terms of the wavevectors. The interference between pump ${\vec{k}}_{L}$ and Stokes waves ${\vec{k}}_{S}$ generates a plane sound wave $\vec{q}={\vec{k}}_{L}-{\vec{k}}_{S}$, providing energy conversion from the pump wave to the Stokes wave. Under assumption of small angles $\alpha_{L},\;\alpha_{L}<<1$, Equation (2) gives the following expressions for the acoustic wave amplitude and the normalized gain factor:

(6) $$\begin{array}{l} p\left(\delta ,{\vec{r}}_{⊥}\right)=\frac{{\vec{e}}_{L}^{*}\left({\vec{r}}_{⊥}\right)\cdot {\vec{e}}_{S}\left({\vec{r}}_{⊥}\right)}{1+i\left(\delta -T_{2}\Omega_{0}\frac{{\left(\alpha_{L}+\alpha_{S}\right)}^{2}}{8}\right)} \end{array}$$

(7) ReGδ,φ=11+δ−Ω0T2αL+αS282

Equations (6) and (7) highlight several features of the SBS interaction in the plane wave pair that are important for further discussion. First, the SBS gain spectrum has a FWHM linewidth $\Delta \nu_{S}\sim 1/\pi T_{2}$ (Δδ~1), determined by the sound relaxation time $T_{2}$.

**Figure 3:**
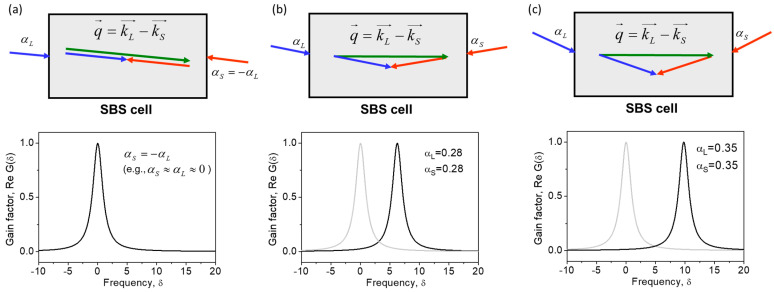
Brillouin gain spectra describing interaction between plane waves in a volume medium at different angles (**a**–**c**). Calculations are performed using Equation (7).

Second, the peak Brillouin gain factor does not depend on the plane wave interaction angles, but its position in the gain spectrum does. At $\alpha_{L}=-\alpha_{S}$, the interacting waves are strongly counterpropagating and the SBS gain spectrum is most shifted to the Stokes side ($\delta_{0}=0$). With an increase in the interaction angle $\alpha_{L}+\alpha_{S}$, the SBS gain line simply shifts to the anti-Stokes side, obtaining $\delta_{0}=\Omega_{0}T_{2}\frac{{\left(\alpha_{L}+\alpha_{S}\right)}^{2}}{8}$. This feature is illustrated in Figure 3.

Finally, one can see from Equation (6) that $p\left(\delta ,{\vec{r}}_{⊥}\right)\sim {\vec{e}}_{L}^{*}\left({\vec{r}}_{⊥}\right)\cdot {\vec{e}}_{S}\left({\vec{r}}_{⊥}\right)$ at any δ. This means that the acoustic grating recorded by the interference of two optical modes propagates through the medium, maintaining permanent resonance with its parent interference pattern, thus enabling the maximal possible efficiency of the SBS interaction at the given δ.

### 3.2. SBS Interaction between Two Modes in a Planar Waveguide

**Figure 4:**
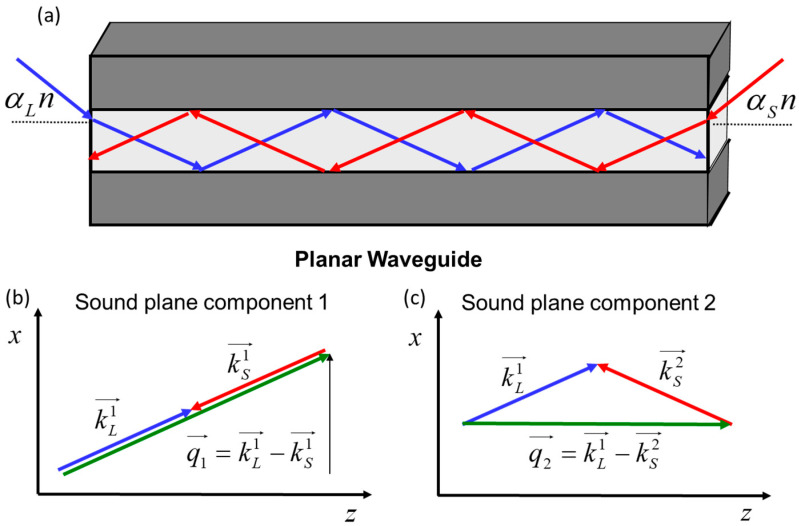
Brillouin interaction between eigen optical modes in a planar waveguide (**a**). Each optical mode is a combination of two plane waves, resulting in two kinds of sound plane waves possessing low (**b**) and high (**c**) spatial modulation frequencies in the waveguide cross-section.

The SBS amplification process in an optical waveguide differs from the SBS in a volume medium. Even in the simplest case of a planar waveguide considered in this section, the introduced optical waves experience reflections from the waveguide boundaries, resulting in a complex interference pattern that could be expressed as a superposition of the waveguide eigenmodes. Such an eigenmode is superposed from two plane waves with wavevectors ${\vec{k}}_{}^{1}$ and ${\vec{k}}_{}^{2}$, possessing the reflection symmetry relative to plane y = 0. The eigen optical modes ${\vec{e}}_{L}\left({\vec{r}}_{⊥}\right)$, ${\vec{e}}_{S}\left({\vec{r}}_{⊥}\right)$ are reduced to:

(8) $$\begin{array}{l} {\vec{e}}_{L}\left(x\right)=\left\{\cos\left(k_{⊥L}x\right);\;\;\sin\left(k_{⊥L}x\right)\right\}\\ {\vec{e}}_{S}\left(x\right)=\left\{\cos\left(k_{⊥S}x\right);\;\;\sin\left(k_{⊥S}x\right)\right\} \end{array}$$

where $k_{⊥L,S}=k_{L,S}\sin\alpha_{L,S}$, $-x_{d}<x<x_{d}$ is the transverse coordinate in the waveguide with the thickness $2x_{d}$, and $\alpha_{L}^{\pm }$ and $\alpha_{S}^{\pm }$ are the plane wave incidence angles. To excite an individual mode, their incidence angles $\alpha_{i}^{\pm }$ and $-\alpha_{i}^{\pm }$ should be taken from the set of discrete values $\left\{\alpha_{i}^{\pm }\right\}$ to match the boundary conditions at the waveguide walls (at $x=\pm x_{d}$), which for multimode waveguide are ${\vec{e}}_{L,S}\left(\pm x_{d}\right)\approx 0$. The Brillouin interaction between the pump and Stokes optical modes could be thought of as an interaction between four plane waves driven by pump ${\vec{k}}_{L}^{1}$, ${\vec{k}}_{L}^{2}$ and Stokes ${\vec{k}}_{S}^{1}$, ${\vec{k}}_{S}^{2}$ wavevectors, as is illustrated in Figure 4 (both pairs of wavevectors are symmetric with respect to the plane x=0). These plane waves interact with each other and could produce four sound plane waves: ${\vec{q}}_{1}={\vec{k}}_{L}^{1}-{\vec{k}}_{S}^{1}$, ${\vec{q}}_{2}={\vec{k}}_{L}^{1}-{\vec{k}}_{S}^{2}$, and symmetric ones, ${\vec{q}}_{\tilde{1}}={\vec{k}}_{L}^{2}-{\vec{k}}_{S}^{2}$, ${\vec{q}}_{\tilde{2}}={\vec{k}}_{L}^{2}-{\vec{k}}_{S}^{1}$. Under assumption of small angles $\alpha_{L},\;\alpha_{S}<<1$ the acoustic wave amplitude $p\left(\delta ,\vec{r}\right)$ is expressed from Equation (4) as a superposition of two pairs of sound plane waves:

(9) $$p\left(\delta ,\vec{r}\right)\sim \frac{\exp\left[\pm i\left|{\vec{q}}_{1⊥}\right|x\right]}{1\pm i\left(\delta -T_{2}\Omega_{0}\frac{{\left(\alpha_{L}-\alpha_{S}\right)}^{2}}{8}\right)};\;\sim \;\frac{\exp\left[\pm i\left|{\vec{q}}_{2⊥}\right|x\right]}{1\pm i\left(\delta -T_{2}\Omega_{0}\frac{{\left(\alpha_{L}+\alpha_{S}\right)}^{2}}{8}\right)}$$

In the waveguide cross-section plane, the first and second sound plane waves exhibit different spatial modulation frequencies, i.e., high $\left|{\vec{q}}_{1⊥}\right|=\left(\left|k_{⊥L}\right|+\left|k_{⊥S}\right|\right)$ and low $\left|{\vec{q}}_{2⊥}\right|=\left(\left|k_{⊥L}\right|-\left|k_{⊥S}\right|\right)$ spatial frequencies, respectively, where $k_{⊥L,S}=\left|k_{L,S}\right|\sin\alpha_{L,S}$. Note that the first of them possesses Brillouin resonance at low (more Stokes side shifted) temporal frequency of $\delta_{1}=T_{2}\Omega_{0}\frac{{\left(\alpha_{L}-\alpha_{S}\right)}^{2}}{8}$, whereas the second possesses Brillouin resonance at a higher temporal frequency of $\delta_{2}=T_{2}\Omega_{0}\frac{{\left(\alpha_{L}+\alpha_{S}\right)}^{2}}{8}$. Until the incident angles $\alpha_{L},\alpha_{S}$ are small (i.e., $\frac{\Omega_{0}T_{2}{\left(\left|\alpha_{L}\right|+\left|\alpha_{S}\right|\right)}^{2}}{8}\;<<1$), all optical wavevectors are almost collinear with *z*, and the corresponding sound waves degenerate to a single plane wave, with the wavevector $\vec{q}$ parallel to *z*. An increase in the incident angles $\alpha_{L},\alpha_{S}$ causes inhomogeneous broadening and splitting of the SBS gain spectrum due to the different resonances in different plane wave pairs.

The exact expression for the SBS gain spectrum reads as:

(10) ReGδ,φ=1211+δ−Ω0T2αL−αS282+1211+δ−Ω0T2αL+αS282

**Figure 5:**
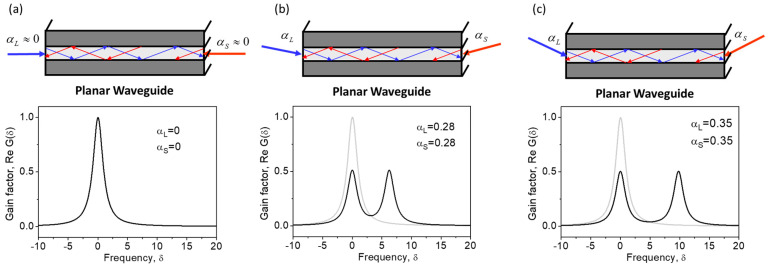
Brillouin gain spectra for the interaction between eigenmodes of different orders (**a**–**c**) in a planar waveguide. Calculations are performed using Equation (10).

Figure 5 shows the broadening and splitting of the SBS gain spectrum with an increase in incidence angles. At large incidence angles, the SBS spectrum exhibits two identical peaks corresponding to the pump scattering by two sound waves. The right peak at $\delta =\delta_{2}$ is the same as in the case of the plane wave interaction in a volume medium. It is associated with pump scattering by a sound wave possessing a lower spatial frequency $\left|{\vec{q}}_{2⊥}\right|$ in the waveguide cross-section (Figure 4c). The left peak at a much smaller $\delta =\delta_{1}$ is associated with pump scattering by a sound wave with a higher spatial frequency $\left|{\vec{q}}_{1⊥}\right|$(Figure 4b). We can therefore conclude that the appearance of the left peak at $\delta =\delta_{1}$ is caused by the guiding manner of the light propagation in the optical waveguide. Note that at $\alpha_{L}\approx \alpha_{S}$, the additional peak is located near the central Stokes frequency $\delta_{1}\approx 0$ ($\omega_{S}\approx \omega_{S0}$).

## 4. SBS Interaction between Individual Modes in an Optical Fiber

### 4.1. Weakly Guiding Modes

To describe SBS interaction between individual optical modes in an optical fiber, based on Equations (1)–(4), the explicit expressions for fiber modes ${\vec{e}}_{L}\left(r,\varphi \right)$, ${\vec{e}}_{S}\left(r,\varphi \right)$ should be specified. Hereafter, we use the approximation of weakly guiding step-index cylindrical fibers ($\Delta =1-n_{core}/n_{clad}<<1$, where $n_{core}$, $n_{clad}$ are the refractive index of the core and cladding) [37]. There are $\sim V^{2}/2$ guided modes that are characterized by their own orbital l and radial p parameters for the optical fiber with a numerical aperture NA, a core radius a and parameter $V=2\pi \frac{a}{\lambda }\;NA$. At l=0, for each $i=\left\{0,p\right\}$, there are two modes:

1. Even modes $HE_{1,p}$: ${\vec{e}}_{1,\;0,p}\left({\vec{r}}_{}\right)=\vec{x}J_{l}\left(u_{l}^{1,p}r\right)$
2. Odd modes $HE_{1,p}$: ${\vec{e}}_{3,\;0,p}\left({\vec{r}}_{}\right)=\vec{y}J_{l}\left(u_{l}^{3,p}r\right)$

At l≥1, for each $i=\left\{l,p\right\}$, there are four modes:

1. Even modes $HE_{l+1,p}$: ${\vec{e}}_{1,\;l,p}\left({\vec{r}}_{⊥}\right)=\left\{\vec{x}\cos\left(l\varphi \right)-\vec{y}\sin\left(l\varphi \right)\right\}J_{l}\left(u_{l}^{1,p}r\right)$
2. Even modes $EH_{l-1,p}$: ${\vec{e}}_{2,\;l,p}\left({\vec{r}}_{⊥}\right)=\left\{\vec{x}\cos\left(l\varphi \right)+\vec{y}\sin\left(l\varphi \right)\right\}J_{l}\left(u_{l}^{2,p}r\right)$
3. Odd modes $HE_{l+1,p}$: ${\vec{e}}_{3,\;l,p}\left({\vec{r}}_{⊥}\right)=\left\{\vec{x}\sin\left(l\varphi \right)+\vec{y}\cos\left(l\varphi \right)\right\}J_{l}\left(u_{l}^{3,p}r\right)$
4. Odd modes $EH_{l-1,p}$: ${\vec{e}}_{4,\;l,p}\left({\vec{r}}_{⊥}\right)=\left\{\vec{x}\sin\left(l\varphi \right)-\vec{y}\cos\left(l\varphi \right)\right\}J_{l}\left(u_{l}^{4,p}r\right)$

Here, $J_{l}\left(u_{l}^{s,p}r\right)$ denotes Bessel functions to be a solution of the characteristic equations $uJ_{l+1}\left(u\right)/J_{l}\left(u\right)=\pm wK_{l+1}\left(w\right)/K_{l}\left(w\right)$, where $w=\sqrt{V^{2}-u^{2}}$ gives sets of radial phase parameters $u_{l}^{1,p}\equiv u_{l}^{3,p}$ (sign +) and $u_{l}^{2,p}\equiv u_{l}^{4,p}$ (sign -), where p is the ordinal number of the solution in ascending order.

All even modes have different propagation constants, $\beta_{l}^{1,p}$ and $\beta_{l}^{2,p}$. All odd modes have the same propagation constants as the corresponding even modes. At l=1, the propagation constants for all modes are different. The propagation constants $\beta_{l}^{1,p}$ and $\beta_{l}^{2,p}$ differ by ~Δ32βlp. We can specify the modes as $m=\left\{l,p,s\right\}$, where s=1...4 is the type of the mode in the given classification.

### 4.2. Derivation of Expressions for Acoustic Wave Amplitude and Brillouin Gain Spectrum

Now we have to substitute the expressions for pump and Stokes optical modes into Equation (4) to solve it analytically. The pump and Stokes modes are determined by the sets of indexes $L=\left\{l_{L},p_{L},s_{L}\right\}$ and $S=\left\{l_{S},p_{S},s_{S}\right\}$, respectively, where $l_{L}$, $l_{S}$ and $p_{L}$, $p_{S}$ are orbital and radial optical mode parameters, and $s_{L}$,$s_{S}$ set the type of the mode. Considering the interaction between two arbitrary pump and Stokes modes, we denote $u_{L}=u_{l_{L}}^{s_{L},p_{L}}$,$u_{S}=u_{l_{S}}^{s_{S},p_{S}}$ and express the scalar product $\left({\vec{e}}_{L}^{*}\cdot {\vec{e}}_{S}\right)=J_{l_{L}}\left(u_{L}r\right)J_{l_{S}}\left(u_{S}r\right)f\left(\varphi \right)$, where $f\left(\varphi \right)$ is the function defined in Table 1 for modes of odd and even types. The sign (+ or -) between $l_{L}$ and $l_{S}$ in the expression for $f\left(\varphi \right)$ is important, so we distinguish $f^{-}\left(\varphi \right)$ and $f^{+}\left(\varphi \right)$. Then, using $p\left(r,\varphi \right)=\rho \left(r\right)f\left(\varphi \right)$, we separate the variables in Equation (4), thus obtaining the equation describing the radial distribution of sound wave amplitude $\rho \left(r\right)$:

(11) $$\begin{array}{l} \left(1+i\left(\delta -\delta_{LS}\right)\right)\rho \left(\delta ,r\right)-\\ \;\;\;\;\;\;-i\mu \;\left[\frac{d^{2}}{dr^{2}}+\frac{1}{r}\frac{d}{dr}-\frac{{\left(l_{L}\pm l_{S}\right)}^{2}}{r^{2}}\right]\rho \left(\delta ,r\right)=J_{l_{L}}\left(u_{L}r\right)J_{l_{S}}\left(u_{S}r\right) \end{array}$$

where the sign in $l_{L}\pm l_{S}$ is taken as in $f^{\pm }\left(\varphi \right)$.

Now, we are looking for the solution of Equation (11) as a superposition of solutions for the corresponding homogeneous equation:

(12) $$\rho \left(\delta ,r\right)=\int D\left(\lambda \right)J_{l_{L}\pm l_{S}}\left(\sqrt{\lambda r}\right)d\lambda$$

where $J_{l_{S}\pm l_{S}}$ denotes the Bessel function and $D\left(\lambda \right)$ is an unknown function to be defined.

The function $J_{l_{L}\pm l_{S}}\left(\sqrt{\lambda r}\right)$ could be expanded in series using the Graf’s Addition Theorem [38]:

(13) $$\begin{array}{cc} J_{s}\left(\sqrt{\lambda }r\right) & =\frac{1}{\cos\left(s\chi \left(\lambda \right)\right)}\sum_{k=-\infty }^{+\infty }J_{s+k}\left(ur\right)J_{k}\left(vr\right)\cos\left(k\alpha \left(\lambda \right)\right)=\\ & =\frac{1}{\sin\left(s\chi \left(\lambda \right)\right)}\sum_{k=-\infty }^{+\infty }J_{s+k}\left(ur\right)J_{k}\left(vr\right)\sin\left(k\alpha \left(\lambda \right)\right) \end{array}$$

where $u,\;v,\;\sqrt{\lambda }$ are thought of as sides of a triangle with angles χ and α:

(14) $$\lambda =\left(u^{2}+v^{2}\right)-2uv\cos\alpha$$

In this way $\rho \left(\delta ,r\right)$ is reduced to:

(15) $$\rho \left(\delta ,r\right)=\sum_{k=-\infty }^{+\infty }J_{s+k}\left(u_{L}r\right)J_{k}\left(u_{S}r\right)\int_{0}^{\pi }\left[a\left(\alpha \right)\cos\left(k\alpha \right)+b\left(\alpha \right)\sin\left(k\alpha \right)\right]\;d\alpha$$

Then, substituting Equation (15) into Equation (11) and extracting the functions $a\left(\alpha \right),\;b\left(\alpha \right)$, we can set the coefficients of the products $J_{S+k}\left(u_{L}r\right)J_{k}\left(u_{S}r\right)$ on the left side of Equation (8) to zero, except for $J_{l_{L}}\left(u_{L}r\right)J_{l_{S}}\left(u_{S}r\right)$.

Finally, we come to the solution of Equation (11) in the form of an infinite series:

(16) $$\left[\begin{array}{l} \rho \left(\delta ,r\right)=\sum_{m=-\infty }^{+\infty }C_{m}\left(\delta \right)J_{l_{L}+m}\left(u_{L}r\right)J_{l_{S}+m}\left(u_{S}r\right)\;\;\;for\;f^{\left(-\right)}\left(\varphi \right)\\ \rho \left(\delta ,r\right)=\sum_{m=-\infty }^{+\infty }{\left(-1\right)}^{m}C_{m}\left(\delta \right)J_{l_{L}-m}\left(u_{L}r\right)J_{l_{S}+m}\left(u_{S}r\right)\;for\;f^{\left(+\right)}\left(\varphi \right) \end{array}\right.$$

where the coefficients $C_{m}\left(\delta \right)$ are the functions of the frequency δ:

(17) $$C_{m}\left(\delta \right)=\frac{1}{\pi }\int_{0}^{\pi }\frac{\cos m\alpha \;d\alpha }{\left[1+i\left(\delta -\beta \left(u^{2}+v^{2}\right)-2uv\beta \cos\alpha \right)\right]}$$

The integral in Equation (17) can be replaced by a closed-loop integral and evaluated using the residue theorem [39]. With the notation

(18) $$\begin{array}{l} d\left(\delta \right)=\delta -\beta \left(u^{2}+v^{2}\right)\\ c=uv\beta \end{array}$$

the integral (17) is reduced to

(19) $$C_{m}=\frac{1}{2\pi }\oint_{\left|z\right|=1}\frac{z^{m}}{cz^{2}+\left(i-d\right)+c}dz=\frac{1}{2\pi c}\oint_{\left|z\right|=1}\frac{z^{m}}{\left(z-z_{1}\right)\left(z-z_{2}\right)}dz$$

where the complex roots $z_{1}=ae^{i\phi }$, $z_{2}=a^{-1}e^{-i\phi }$ satisfy the following expressions:

(20) $$\begin{array}{l} \cos\phi =\frac{d}{c}\frac{a}{a^{2}+1}\\ \sin\phi =\frac{1}{c}\;\frac{a}{1-a^{2}}\;for\;a<1;\\ a^{8}-\frac{d^{2}+1}{c^{2}}a^{6}-2\frac{c^{2}-d^{2}+1}{c^{2}}a^{4}-\frac{d^{2}+1}{c^{2}}a^{2}+1=0 \end{array}$$

The single root of Equation (20), $z_{1}=ae^{i\phi }$, lying in the complex plane inside the circle $\left|z\right|=1$, is expressed as:

(21) $$\begin{array}{l} a=\sqrt{\frac{1-\sqrt{x}}{1+\sqrt{x}}}\\ \phi =\arccos\left(\frac{d}{c}\frac{a}{a^{2}+1}\right)\;\mathrm{sign}\left(\frac{1}{c}\;\frac{a}{1-a^{2}}\right) \end{array}$$

where

(22) $$\begin{array}{l} x=\frac{d^{2}+1+\sqrt{D}-4c^{2}}{d^{2}+1+\sqrt{D}+4c^{2}}\\ D=d^{4}+16c^{4}-8c^{2}d^{2}+2d^{2}+8c^{2}+1 \end{array}$$

In these terms, the coefficient $C_{m}\left(\delta \right)$ reads as

(23) $$C_{m}\left(\delta \right)=a^{m}\;\frac{\sqrt{x}\left\{\left[\cos m\phi +xd\sin m\phi \right]+i\left[\sin m\phi -xd\cos m\phi \right]\right\}}{x^{2}d^{2}+1}$$

**Figure 6:**
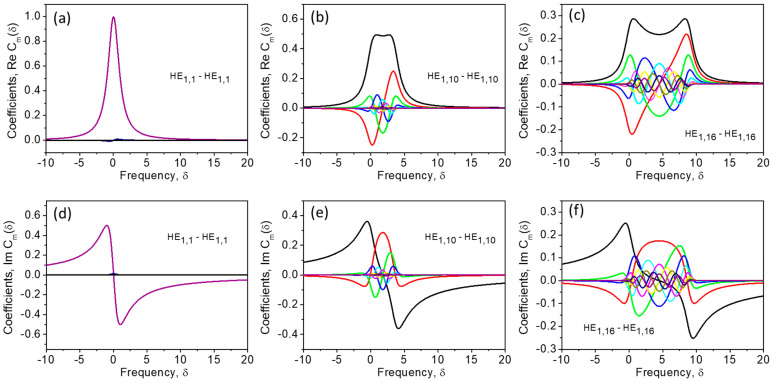
The coefficients $C_{m}\left(\delta \right)$ (m=1,2...10) as functions of the frequency δ for interaction of the modes of different orders (**a**–**f**) in an optical fiber; the real (**a**–**c**) and imaginary (**d**–**f**) parts. Calculations are performed using Equation (23).

Now, substituting Equation (23) into Equation (16) for $\rho \left(r\right)$ and using an appropriate function $f\left(\varphi \right)$ from Table 1, one can obtain an analytical expression describing the distribution of the sound wave amplitude over the fiber cross-section $p\left(r,\varphi \right)=\rho \left(r\right)f\left(\varphi \right)$ for the case of a Brillouin interaction between an arbitrary pair of pump and Stokes optical fiber modes. The azimuthal distribution of sound amplitude is trivial, and is determined by the orbital indices of the interacting modes only. The radial distribution $\rho \left(\delta ,r\right)$ is represented as an infinite sum of Bessel function products with weight coefficients $C_{m}\left(\delta \right)$ (17). Since $C_{m}\left(\delta \right)\sim a^{m}$ and $\left|a\right|<1$, the sum converges. Commonly 10–20 terms are enough to calculate $\rho \left(\delta ,r\right)$ in (16). Figure 6 first demonstrates several coefficients $C_{m}\left(\delta \right)$ in the case of an interaction between the pump and Stokes modes of low (a, d), moderate (b, e) and high (c, f) orders. One can see that in the last case, the series $C_{m}\left(\delta \right)$ converges more slowly.

Now, substituting the expression for $\rho \left(\delta ,r\right)$ and $f\left(\varphi \right)$ into Equation (3), we come to the solution for the SBS gain spectrum in the form:

(24) $$G\left(\delta \right)=\frac{1}{N_{L}N_{S}}K_{\phi }\left(s_{L},s_{S},l_{L},l_{S}\right)\int_{0}^{1}\left[J_{l_{L}}\left(u_{L}r\right)J_{l_{S}}\left(u_{S}r\right)\rho \left(\delta ,r\right)\right]rdr$$

where $N_{L}=\int_{0}^{1}{\left[J_{l_{L}}\left(u_{L}r\right)\right]}^{2}rdr$ and $N_{S}=\int_{0}^{1}{\left[J_{l_{S}}\left(u_{L}r\right)\right]}^{2}rdr$ are normalization coefficients, and

(25) $$K_{\phi }\left(s_{L},s_{S},l_{L},l_{S}\right)=\frac{1}{4\pi }\int_{0}^{2\pi }f_{l_{L},l_{S}}^{2}\left(\phi \right)\;d\phi$$

The values of $K_{\phi }\left(s_{L},s_{S},l_{L},l_{S}\right)$ for interacting modes of different types and orbital moments are presented in Table 2.

Using Equation (23), the Brillouin gain factor (24) can be converted to the following format:

(26) $$G\left(\delta \right)=K_{\phi }\left(s_{L},s_{S},l_{L},l_{S}\right)\sum_{m=-\infty }^{\infty }C_{m}\left(\delta \right)K^{\pm r}\left(l_{L},l_{S},p_{L},p_{S},m\right)$$

where the sign (+) or (-) is chosen as in $f^{\pm }\left(\varphi \right)$, and

(27) $$\begin{array}{l} K^{-r}\left(l_{L},l_{S},p_{L},p_{S},m\right)=\frac{1}{N_{L}N_{S}}\int_{0}^{1}\left[J_{l_{L}}\left(u_{L}r\right)J_{l_{S}}\left(u_{S}r\right)J_{l_{L}+m}\left(u_{L}r\right)J_{l_{S}+m}\left(u_{S}r\right)\right]rdr\\ K^{+r}\left(l_{L},l_{S},p_{L},p_{S},m\right)=\frac{{\left(-1\right)}^{m}}{N_{L}N_{S}}\int_{0}^{1}\left[J_{l_{L}}\left(u_{L}r\right)J_{l_{S}}\left(u_{S}r\right)J_{l_{L}-m}\left(u_{L}r\right)J_{l_{S}+m}\left(u_{S}r\right)\right]rdr \end{array}$$

Note that all coefficients, Equations (25) and (27), are real. They are completely defined by the fiber parameters, and for the given fiber should be tabulated just once.

It worth noting that the coefficients $C_{m}\left(\delta \right)$ accumulate all dependence on the frequency δ. Their linear combinations form both the gain spectrum profile $G\left(\delta \right)$ and the radial distribution $\rho \left(\delta ,r\right)$ as a function of δ. The sum in Equation (26) for the gain profile $G\left(\delta \right)$ converges as fast as the sum in Equation (10), describing the sound amplitude.

## 5. Sound Propagation Effects

One can see from Figure 6 that the number of terms required for precise characterization of the Brillouin process through Equations (16) and (26) depends on the parameter μ that evaluates the strength of the sound propagation effects accompanying the Brillouin amplification process in optical fiber. Indeed, at $\mu \left(u_{L}{}^{2}+u_{S}{}^{2}\right)\to 0$, all coefficients $C_{m}\left(\delta \right)\to 0$, except $C_{0}\left(\delta \right)$, and the spatial distribution of the sound amplitude $p\left(\delta ,{\vec{r}}_{⊥}\right)=\frac{{\vec{e}}_{L}^{*}\left({\vec{r}}_{⊥}\right)\cdot {\vec{e}}_{S}\left({\vec{r}}_{⊥}\right)}{\left(1+i\delta \right)}$, with some weight determined by δ, coincides with the parent interference pattern ${\vec{e}}_{L}^{*}\left({\vec{r}}_{⊥}\right)\cdot {\vec{e}}_{S}\left({\vec{r}}_{⊥}\right)$. As $\mu \left(u_{L}{}^{2}+u_{S}{}^{2}\right)$ increases, more and more neighboring components $\sim J_{l1\pm m}\left(u_{L}r\right)J_{l2+m}\left(u_{S}r\right)$ become significant in the expansion (16), causing a mismatch between the spatial distribution of sound amplitude $\rho \left(\delta ,r\right)$ and the parent interference pattern ${\vec{e}}_{L}^{*}\left({\vec{r}}_{⊥}\right)\cdot {\vec{e}}_{S}\left({\vec{r}}_{⊥}\right)$. This mismatch reduces the efficiency of Brillouin interaction in optical fiber. This is in contrast with the SBS in a planar waveguide that produces the sound amplitude which is always coinciding with the parent interference pattern.

**Figure 7:**
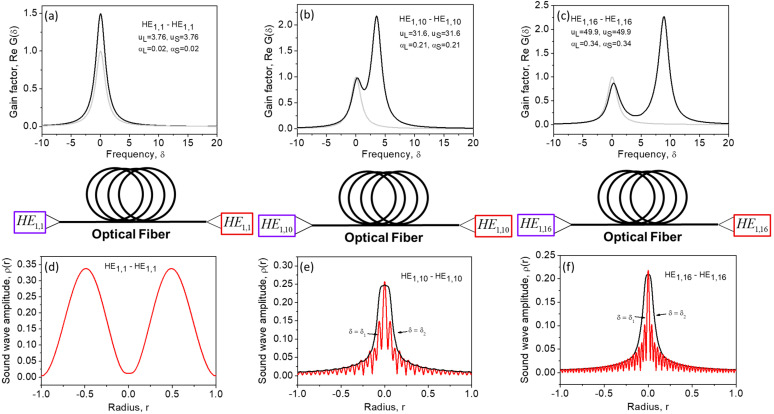
The SBS gain spectra $G\left(\delta \right)$ (**a**–**c**) and radial distribution of the sound amplitude $\rho \left(\delta ,r\right)$ (**d**–**f**) at the frequencies δ corresponding to the left (red curve) and right (black curve) peak of the SBS gain spectra for interaction of modes of different orders (**a**–**f**) in an optical fiber. Calculations are performed using Equations (16), (23), (26) and (27).

Figure 7 shows the SBS gain spectra calculated for different interacting mode pairs (a-c) using Equation (26) and radial distributions of the sound amplitude $\rho \left(\delta ,r\right)$ (d-f) at peak δ values, using Equation (12). At $\mu \left(u_{L}{}^{2}+u_{S}{}^{2}\right)<<1$, in the case of interaction between two low-order modes, the gain spectrum shown in Figure 7a has only one peak at δ=0, with a width of $\Delta \nu_{S}\sim \frac{1}{\pi T_{2}}$ (Δδ~1). A single peak SBS gain spectrum shown in Figure 7a is similar to that shown in Figure 4a for the SBS in a planar waveguide (for small incident angles), but the maximal SBS gain exceeds the SBS factor for a volume medium more than twice. This is due to nonuniform (bell-like) distribution of the pump power in the fiber core which reduces the effective fiber core area available for nonlinear interaction. The radial distribution of the peak sound amplitude at δ=0 is shown in Figure 7d. One can see that it exhibits a low spatial nonuniformity, but it is not a purely uniform distribution as in the case of the planar waveguide (for small incident angles, when the sound plane wave wavevector is parallel to *z*).

In the case of interaction between two high-order modes (b, c) the SBS gain spectrum exhibits two peaks. The peak observed at higher frequency $\delta_{2}=\mu {\left(u_{L}+u_{S}\right)}^{2}$ is associated with pump scattering from the sound wave component $\rho \left(\delta_{2},r\right)$, possessing lower transverse inhomogeneity (e, f, black curves). The peak of the SBS gain spectrum at the lower frequency $\delta_{1}=\mu {\left(u_{L}-u_{S}\right)}^{2}$ is associated with scattering from the sound wave component $\rho \left(\delta_{1},r\right)$, possessing higher transverse inhomogeneity (e, f, red curves). These features are similar to that reported for the sound waves with high ${\vec{q}}_{1⊥}$ and low ${\vec{q}}_{2⊥}$ spatial frequencies in the case of a planar waveguide shown in Figure 4. However, in contrast to the case of planar waveguide, two peaks of the SBS gain spectrum in an optical fiber possesses different amplitudes. The SBS gain peak at $\delta_{2}=\mu {\left(u_{L}+u_{S}\right)}^{2}$ is always higher than that at $\delta_{1}=\mu {\left(u_{L}-u_{S}\right)}^{2}$. This specific feature is attributed to the sound propagation effect illustrated in Figure 6. At lower frequency $\delta =\delta_{1}$, the SBS interaction is governed by the sound wave component with higher transverse inhomogeneity that acquires a stronger mismatch with the copropagating parent interference pattern ${\vec{e}}_{L}^{*}\left(\vec{r}\right)\cdot {\vec{e}}_{S}\left(\vec{r}\right)$, thus reducing the efficiency of Brillouin process. This is in contrast to the SBS in a planar waveguide wherein the sound amplitude always coincides with the parent interference pattern, and both peaks of the SBS gain spectrum are of equal amplitudes.

**Figure 8:**
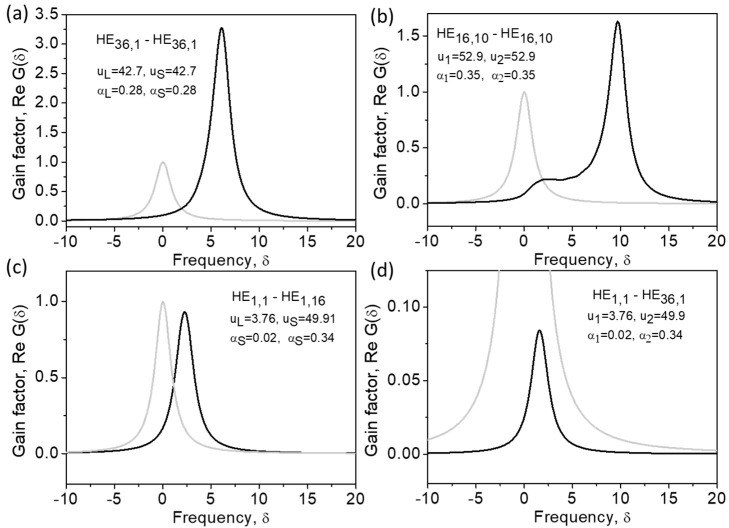
Brillouin gain spectra in the cases of interaction between eigen modes of different orders (**a**–**d**) in an optical fiber. Calculations are performed using Equations (26) and (27).

Figure 8 shows more examples of the Brillouin gain spectra demonstrating these features. One can see that the efficiency of Brillouin interactions decreases with an increase in the orbital numbers of the interacting modes (e), especially in the case in which the orbital numbers of the interacting modes are different (g).

## 6. Discussion

Due to sound propagation effects described in the previous sections, the SBS amplification process in an optical waveguide differs from the SBS in a volume medium. The difference is most pronounced when the SBS process is between two high-order optical modes. In this case, the resonant Brillouin interaction could be achieved with two different sound wave components induced at two different resonance frequencies, and as a result, the SBS gain spectrum splits, exhibiting two peaks. First, the resonant sound wave with high-frequency spatial modulation in the fiber core is responsible for the formation of the low-frequency spectrum peak. Second, the resonant sound wave with low-frequency spatial modulation in the fiber core is responsible for the formation of the high-frequency spectrum peak. In the planar waveguide, both peaks of the SBS gain spectrum have the same amplitude. In the cylindrical optical fiber, the peaks possess different amplitudes. The amplitude of the low-frequency peak is always lower than the amplitude of the high-frequency peak. This feature is attributed to the specific property of the SBS amplification process considered in the previous section, which could be referred to as the sound diffraction effect.

This term recalls the sound diffraction effect widely discussed in the past in the context of SBS in single-mode optical fibers [40,41,42,43]. Indeed, in a single-mode fiber, the sound wave is generated in the fiber core, where the light is localized. So, the sound wave is generated within a small transverse fiber area with the size of $a\sim \lambda_{L}$ (comparable with the sound wave wavelength $\sim \lambda_{L}/2n$), and suffers diffraction divergence as it propagates in the fiber. As a result, it runs away from the fiber core, impairing the efficiency of its interaction with the optical fields. This process is important, and affects the SBS process when the time associated with the sound divergence $\tau_{D}\approx a^{2}/v\lambda_{L}$ becomes smaller than the sound relaxation time $T_{2}$: $\tau_{D}<T_{2}$. In other words, the diffraction time constant $\tau_{D}$ becomes significant and replaces $T_{2}$ in the 1-D SBS dynamical equations and expressions for the Brillouin gain spectrum [11,35,44], causing its broadening and thereby suppressing the Brillouin interaction in a single-mode fiber.

In this paper, we have demonstrated that a similar effect could be obtained with the SBS in multimode fibers. When the SBS involves interaction of high-order optical modes, the sound diffraction effect occurs due to the different manner of propagation in the optical fiber of optical and sound waves. The optical fiber supports the waveguide manner of propagation for optical waves only, whereas it remains a volume medium for sound waves (until we ignore its guiding and anti-guiding properties at sound frequencies). The optical eigenmodes in an optical fiber are expressed through special functions, while the sound eigenmodes remain to be plane waves. As a result, a sound wave generated in some fiber points by the interference pattern produced by a pair of pump and Stokes eigenmodes ${\vec{e}}_{L}\left({\vec{r}}_{⊥}\right)$, ${\vec{e}}_{S}\left({\vec{r}}_{⊥}\right)$ has a transverse structure $\sim \left\{{\vec{e}}_{L}{}^{*}\left({\vec{r}}_{⊥}\right){\vec{e}}_{S}^{}\left({\vec{r}}_{⊥}\right)\right\}$ that is not maintained during its further free propagation through the fiber. The mismatch between the sound wave and traveling interference pattern occurs with the typical time $\tau_{D}=\frac{n}{c}\frac{4\pi a^{2}}{\lambda_{L}\left(u_{L}{}^{2}+u_{S}{}^{2}\right)}$. When this mismatch occurs faster than the sound wave decays $\tau_{D}≻T_{2}$, the sound diffraction effect takes charge for the SBS gain spectrum broadening, resulting in suppression of the SBS interaction near the low-frequency spectral peak. In contrast, the sound diffraction effect is not observed with the planar (and rectangle) waveguides, since both optical and sound eigenmodes are plane waves. In this case, a sound wave generated by the interference between pump and Stokes eigenmodes always keeps its resonance with the parent interference pattern, as they both propagate through the fiber.

## 7. Conclusions

In conclusion, we have studied the SBS interaction in optical fiber implemented with a pair of counter-propagating optical modes. In contrast to the previously reported theoretical considerations [28,29,30], we use a weakly guided optical fiber model and have managed to build analytical expressions for the SBS gain spectrum (Equations (26) and (27)) and sound wave core profile (Equations (16) and (23)) eligible for the SBS interaction between two arbitrary modes. To obtain analytical expressions, we have applied Graf’s addition theorem [38] to the integral describing the sound cross-section profile (Equation (12)) and then used the residue theorem [39], resulting in the further conversion of the integral into a simple, rapidly converging series. In this series, only a limited number of terms determine the properties of the defined functions, making their relations with the specific mutual dynamics of light and sound waves in multimode optical fibers obvious.

To the best of our knowledge, we have described for the first time the sound diffraction effect for SBS in multimode optical fibers, which is similar to that known earlier for SBS in single-mode fibers only. To expose the nature of the effect, the SBS in fibers with cylindrical symmetry has been compared with the SBS in a volume medium and planar waveguides wherein the sound diffraction effect is not supported. In this way, we have explored the splitting of the SBS gain spectrum determined by the waveguide character of the optical light propagation in the optical fiber, and analyzed the features of the SBS gain spectrum broadening.

It is worth noting that the developed approach could be extended to describe the SBS interaction between groups of modes selectively excited in multimode optical fibers, thus enabling a simplified analysis of the mode conversion processes (including the OPC effect) performed immediately in multimode optical fibers. Additionally, a similar mathematical treatment could be applied to other SBS models employing the descriptions of optical fiber modes expressed through Bessel functions.

Looking to future experiments that have to be performed to verify the theoretical predictions reported in this work, the amplified narrow-band fiber lasers [45,46,47,48,49,50] combined with the all-digital hologram and phase plate devices [51,52] could be considered as valuable candidates to serve as critical elements of the experimental setup, enabling the selective excitation of pure single optical modes in multimode fibers, and their de-multiplexing at the fiber output. Direct control of the optical field amplitudes and phases through a flexible SLM used as a holographic filter enables a fast switch of the excited fiber mode composition. Combining this procedure with a mode-analyzing technique allows the evaluation of the excited mode purity. A feedback control system between the mode analysis and the mode excitation would be essential to minimize the mode excitation errors and compensate for distortions caused by the fiber environment.

We believe our findings will stimulate progress in the significant drive to develop modern imaging and mode-division multiplexing sensor techniques, as discussed in the introduction. In particular, using the properties of the SBS gain spectrum similar to that shown in Figure 7 and Figure 8, the SBS could supply these techniques by selective mode amplification and suppression, resulting in direct optical mode processing performed immediately in multi-mode optical fibers. In addition, this could enable new sensing applications of the optical Vernier effect through employing slightly detuned Brillouin frequency shifts that are naturally implemented to optical modes of different orders, since this is an inherent property of the SBS in optical fiber (Equations (26) and (27)).

## Figures and Tables

**Table 1:** The function $f\left(\varphi \right)$ for different types of pump/Stokes modes used for SBS interaction.

Pump\Stokes	$1.\;\mathrm{Even}\;\mathrm{Mode}\;HE_{l+1,p}$	$2.\;\mathrm{Even}\;\mathrm{Mode}\;EH_{l-1,p}$	$3.\;\mathrm{Odd}\;\mathrm{Mode}\;HE_{l+1,p}$	$4.\;\mathrm{Odd}\;\mathrm{Mode}\;EH_{l-1,p}$
1. Even mode $HE_{l+1,p}$	$\cos\left(l_{L}-l_{S}\right)\varphi$	$\cos\left(l_{L}+l_{S}\right)\varphi$	$-\sin\left(l_{L}-l_{S}\right)\varphi$	$\sin\left(l_{L}+l_{S}\right)\varphi$
2. Even mode $EH_{l-1,p}$	$\cos\left(l_{L}+l_{S}\right)\varphi$	$\cos\left(l_{L}-l_{S}\right)\varphi$	$\sin\left(l_{L}+l_{S}\right)\varphi$	$-\sin\left(l_{L}-l_{S}\right)\varphi$
3. Odd mode $HE_{l+1,p}$	$\sin\left(l_{L}-l_{S}\right)\varphi$	$\sin\left(l_{L}+l_{S}\right)\varphi$	$\cos\left(l_{L}-l_{S}\right)\varphi$	$-\cos\left(l_{L}+l_{S}\right)\varphi$
4. Odd mode $EH_{l-1,p}$	$\sin\left(l_{L}+l_{S}\right)\varphi$	$\sin\left(l_{L}-l_{S}\right)\varphi$	$-\cos\left(l_{L}+l_{S}\right)\varphi$	$\cos\left(l_{L}-l_{S}\right)\varphi$

**Table 2:** The coefficient $4K_{\phi }\left(s_{L},s_{S},l_{L},l_{S}\right)$ for different types of interacting modes.

Pump\Stokes	$1.\;\mathrm{Even}\;\mathrm{Mode}\;HE_{l+1,p}$	$2.\;\mathrm{Even}\;\mathrm{Mode}\;EH_{l-1,p}$	$3.\;\mathrm{Odd}\;\mathrm{Mode}\;HE_{l+1,p}$	$4.\;\mathrm{Odd}\;\mathrm{Mode}\;EH_{l-1,p}$
1. Even mode $HE_{l+1,p}$	$1+\delta_{l_{L},l_{S}}$	1	$1-\delta_{l_{L},l_{S}}$	1
2. Even mode $EH_{l-1,p}$	1	$1+\delta_{l_{L},l_{S}}$	1	$1-\delta_{l_{L},l_{S}}$
3. Odd mode $HE_{l+1,p}$	$1-\delta_{l_{L},l_{S}}$	1	$1+\delta_{l_{L},l_{S}}$	1
4. Odd mode $EH_{l-1,p}$	1	$1-\delta_{l_{L},l_{S}}$	1	$1+\delta_{l_{L},l_{S}}$

## Data Availability

Not applicable.
